# Navel orange peel essential oil inhibits the growth and progression of triple negative breast cancer

**DOI:** 10.1186/s12906-024-04525-y

**Published:** 2024-06-14

**Authors:** Chao Yang, Wenwen Zhang, Shi Xiang, Lai Chen, Jiong Chun, Hui Chen

**Affiliations:** 1 Oncology Research Center, Jiangxi Provincial Key Laboratory of Traditional Chinese Medicine Diagnosis and Rehabilitation of Malignant Tumors, Jiangxi University of Chinese Medicine, Nanchang, 330004 China; 2 College of Life Sciences, National Navel Orange Engineering Research Center, Gannan Normal University, Ganzhou, 341000 China

**Keywords:** Navel orange, Essential oil, Triple negative breast cancer, RNA-seq, Apoptosis

## Abstract

**Background:**

Triple Negative Breast Cancer (TNBC) is a particular type of breast cancer with the highest mortality rate. Essential oils are concerned more and more as potential anti-cancer drugs.

**Methods:**

TNBC cells were treated with different concentrations of navel orange peel essential oil (NOPEO), and then a variety of  experiments were performed to investigate the changes in the growth and progression of TNBC cells. MTT assay was performed to detect the proliferation of TNBC cells. The changes of cell cycle and apoptosis were analyzed by FACS. In order to explored the migration of TNBC cells, scratch wound assay was carried out. Western blotting and qPCR were used to examine the expression of proteins and mRNA of related genes. Furthermore, RNA-seq was used to analyze the altered genes and explored the possible signal pathway.

**Results:**

NOPEO demonstrated dose- and time-dependent suppression of TNBC cell growth. TNBC cells showed an increased percentage of G2/M-phase cells and the protein levels of CyclinB1 and CyclinD1 were decreased after NOPEO treatment. The apoptotic cells were increased in the NOPEO treated TNBC cells. The migration mobility was significantly inhibited by NOPEO. In total, 1376 genes were found to be up-regulated and 1335 genes were down-regulated after NOPEO treatment. According to KEGG and GO pathways, the differentially expressed genes were related to MAPK, Jak/stat and FoxQ signaling pathways.

**Conclusion:**

This investigation explored the bio-activity and molecular mechanisms of NOPEO against TNBC cells. These results indicated that NOPEO could suppress TNBC growth and migration perhaps via the MAPK and Jak/stat signaling pathways, which may provide theoretical reference for anticancer drug development. NOPEO may be a potential natural product for the chemotherapeutic of TNBC.

**Supplementary Information:**

The online version contains supplementary material available at 10.1186/s12906-024-04525-y.

## Background

Breast cancer is the second leading cause of cancer death in women and the most prevalent cancer in women in worldwide [1]. Breast cancer segmentation is based on the levels of human epidermal growth factor receptor 2 (HER2), progesterone receptor (PR) and estrogen receptor (ER) by immunohistochemical staining [2]. TNBC is a distinct subtype of breast cancer distinguished by the absence of HER2, ER and PR expression. It is responsible for 15% of all breast cancers and has a poorer prognostic value and survival [3]. Conventional treatments for early TNBC include surgery followed by adjuvant chemotherapy to reduce the risk of the cancer returning. Despite chemotherapy being a common treatment strategy for TNBC, the toxic and mutagenic nature of these agents induces DNA damage in normal cells, leading to death or transformation of non-tumor cells [4, 5]. Therefore, the search for chemotherapeutic agents with fewer collateral effects remains crucial for effective cancer therapy.

The use of natural products has gained considerable attention and is an important part of chemo-prevention of disease by reducing the incidence of adverse events. Essential oil (EO), a diverse group of natural products extracted from plants, possess bio-active properties including antioxidant, anti-mutagenic, anti-depressant, antibacterial, anti-inflammatory, antiviral and anti-cancer properties [6–8]. More and more evidences show the prospects of EO as therapeutic agents for a variety of diseases. Citrus EO is rich in a large number of chemicals and widely used in food and perfume industry [9, 10]. An important way of increasing the economic value of the crop is to exploit citrus EO. Recently, the bio-activity of citrus EO is getting more and more attention, especially in the field of chemo-prevention and treatment of cancer [11, 12]. With IC_50_ values of 87.17 μg/mL (C. pyriformis), 25.91 μg/mL (C. hystrix), 20.41 μg/mL (C. microcarpa) and 11.66 μg/mL (C. limon), the citrus EOs showed potent anti-proliferative activities against HeLa cells [13]. By reducing protein levels of COX-2 and IL-6, Palestinian sweet lime EO could promote apoptosis and suppress inflammation against SW480 cells [14]. The C. medica L. cv. Diamante and C. bergamia EOs were found to be cytotoxic to melanoma A375 cells showing IC_50_ values of 89.1 and 79.3 µg/mL, respectively [15]. In the MTT experiment, C. reticulata peel EO significantly inhibited Dalton’s Lymphoma Ascites (DLA) cells. This was followed by nuclear pyknosis, membrane blebbing, apoptotic body formation and DNA damage resulting in apoptosis. In vivo experiments, mice pre-treated with C. reticulata peel extract were significantly (50%) protected from Dalton’s Lymphoma Ascites compared to post-treated mice (33%), without any noticeable toxic symptoms [16].

The navel orange, a member of the Citrus genus of the Rutaceae family, contains a large number beneficial natural products including essential oil, flavonoids, carotenoids and vitamins [17–20]. While chemical constituent and bio-activities of NOPEO have been studied, the anti-cancer effects and molecular mechanisms of NOPEO on TNBC cells have not been well investigated. In this work, we have obtained NOPEO from the peel of navel oranges by cold pressing, and have also used molecular distillation to remove waxes, carotenoids and pesticide residues that could have an impact on the anti-cancer activity. The objective of this research was to clarify the effects of NOPEO against TNBC cells by examining the cell growth and apoptosis as well as the expression of associated proteins. Assessments of the anti-cancer functions of NOPEO were conducted using the MTT and scratch wound assays, while cell proliferation signaling pathways were investigated. The cell cycle was assessed with flow cytometry and the most relevant proteins involved in the different phases of cell cycle were determined by western blot. Our results showed that NOPEO has anti-cancer properties, causing cell cycle block and cell apoptosis mainly by targeting the MAPK and Jak/stat pathways.

## Methods

### Preparation of NOPEO sample

The NOPEO sample was prepared as described by Yang et al. by cold pressing of gannan navel orange peel followed by molecular distillation [21].

### Cell culture

The TNBC cell lines MDA-MB-231 and MDA-MB-453 were obtained from the Stem Cell Bank, Chinese Academy of Sciences (Shanghai, China). The cells were cultured in Leibovits' medium (L-15) containing 10% (v/v) fetal bovine serum (FBS) and maintained at 37 °C.

### MTT assay

The cell suspension (1 × 10^4^ cells/mL) was prepared by digesting TNBC cells with 0.25% trypsin. 96-well plates were seeded with 200 μL of cell suspension and the NOPEO per well for 24, 48 and 72 h. 20 μL MTT reagent (0.5 mg/mL) was pipetted into each well and incubated for 4 h at 37 °C. The culture medium was then discarded, washed with PBS and shaken with 200 μL dimethyl sulfoxide (DMSO). Absorbance at 490 nm was measured for each sample [22]. The experiment was repeated in triplicate. Six replicate wells were used in each experiment.

### Propidium Iodide (PI) staining

The treated cells were harvested and rinsed two times with 1 × PBS. Cells were harvested by spinning at 2000 rpm for 5 min and resuspended in 70% ethanol at 4 °C overnight. The cells were incubated with PI (0.05 mg/mL) plus RNase (0.02 mg/mL) for 30 min at 37 °C in the dark. A FACS Coulter flow cytometer (Beckman Coulter, Fullerton, CA, USA) was used to analyse the DNA content.

### Western blotting

To measure protein levels, prior to electrotransfer to PVDF membrane, the NOPEO-treated cell lysates were resolved by 10% SDS-PAGE. The membrane was sequentially blotting by 5% milk, primary and secondary antibodies. The ECL chromogenic solution was used to detect protein expression signals and the ChemiDoc XRS+ instrument (Bio-Rad Laboratories, Hercules, CA, USA) was used to capture luminescence signals.

### Annexin V/PI assay

The tested cells were harvested by trypsinisation and were spun at 3000 rpm for 5 min. The cell pellets was re-mixed in binding buffer at a concentration of 1 × 10^5^–1 × l0^6^ cells per mL. The experimental procedure was according to the protocol of the Annexin V/PI kit (Beyotime, Shanghai, China). The samples were analyzed by FACS (Beckman Coulter, Fullerton, CA, USA) [23].

### Scratch wound assay

When cells grew to 90% confluence, the cell layer was scratched straight with a pipet tip. NOPEO was added to the cells at different density dissolved in serum-free medium. The serum-free medium containing 0.1% (v/v) DMSO was served as a control. A fluorescence microscope (Leica, Danaher Corporation, United States) was used to photograph all cells at 0 and 24 h. The bright field was used to take graph of wound healing detection.

### RNA-seq and data analysis

The MDA-MB-231 cells were exposed to NOPEO or DMSO for 24 h. Three replicates were performed for each group. The samples were dissolved by Trizol and post to Shanghai Majorbio Bio-Pharm Technology Co., Ltd, China for sequencing. The data was analyzed by R language.

### Real-time fluorescence quantitative PCR assay

The NOPEO or DMSO treated MDA-MB-231 cells were extracted into RNA by Trizol. The total RNA samples were reverse transcripted to cDNA by PrimeScript™ RT reagent Kit with gDNA Eraser (Takara). Real-time fluorescence quantitative PCR (qPCR) was performed in triplicate using TB Green® Fast qPCR Mix (Takara Biomedical Technology, Beijing, China). The primers for the RT-PCR are included in Supplementary data S1.

#### Statistical analysis

The Statistical Package for the Social Sciences (SPSS) 20.0 software was applied to analyse the experimental data, which is presented as the mean ± standard deviation (SD) of at least 3 separate experiments.

## Results

### NOPEO inhibited the growth of TNBC

**Fig. 1:**
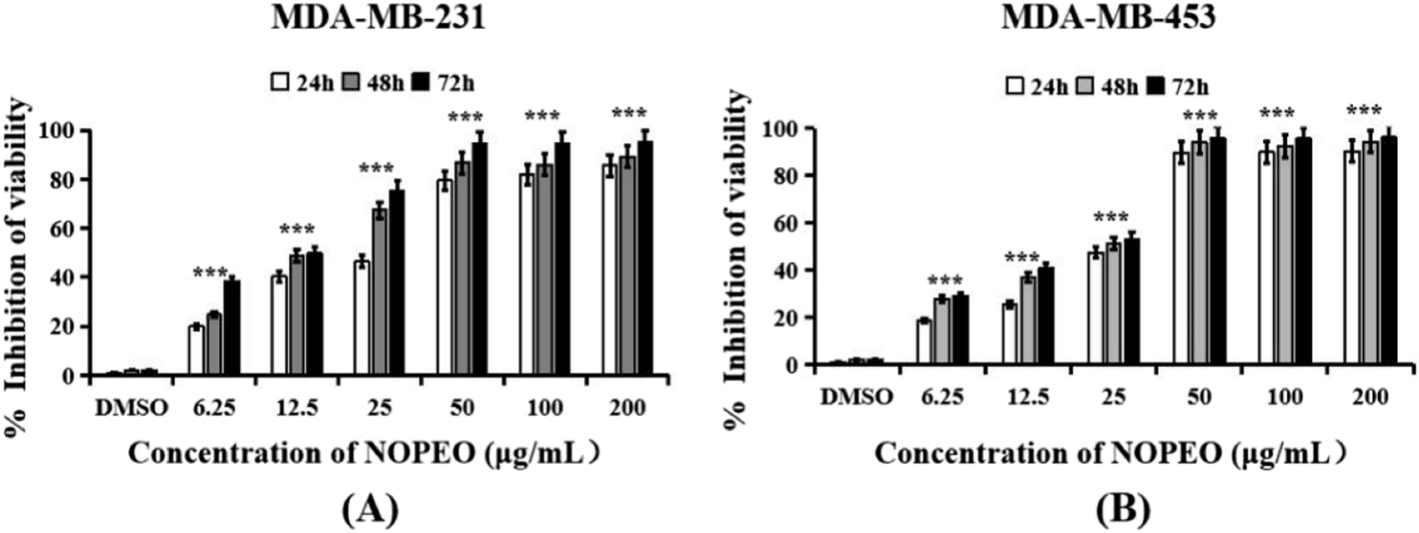
NOPEO inhibited TNBC cells’ proliferation. The inhibition viability of MDA-MB-231 (**A**) and MDA-MB-453 (**B**) cells were cultured with 6.25–200 μg/mL NOPEO and evaluated by the MTT assay

The MTT trial was performed after treatment of MDA-MB-231 and MDA-MB-453 cells with NOPEO at various concentrations to assess the anticancer ability of NOPEO. In Fig. 1, NOPEO demonstrated dose and time-dependent suppression of TNBC cell growth. Viability was inhibited from 19.86 ± 0.79% to 85.52 ± 2.78% for MDA-MB-231 cells (Fig. 1A) and from 18.70 ± 0.83% to 90.46 ± 1.69% for MDA-MB-453 cells (Fig. 1B) by NOPEO treatment (6.25–200 µg/mL) for 24 h. As the treatment time increased from 24 to 72 h, the inhibition rates of cell viability also increased. For example, the percentage of cell viability varied from 19.86 ± 0.79% to 38.31 ± 0.89% when 6.25 μg/mL NOPEO was added to MDA-MB-231 cells from 24 to 72 h.

After 24 h of treatment with NOPEO, the IC_50_ values in MDA-MB-231 and MDA-MB- 453 cells were 22.66 ± 0.68 μg/mL and 23.18 ± 0.76 μg/mL, respectively. Hiva Alipanah et al. found that the IC_50_ values for Citrus sinensis and Citrus limon EO in MDA-MB-468 cells were 23.65 and 40.32 µg/ml, respectively [24].

### NOPEO induced the cell cycle block in TNBC cells

**Fig. 2:**
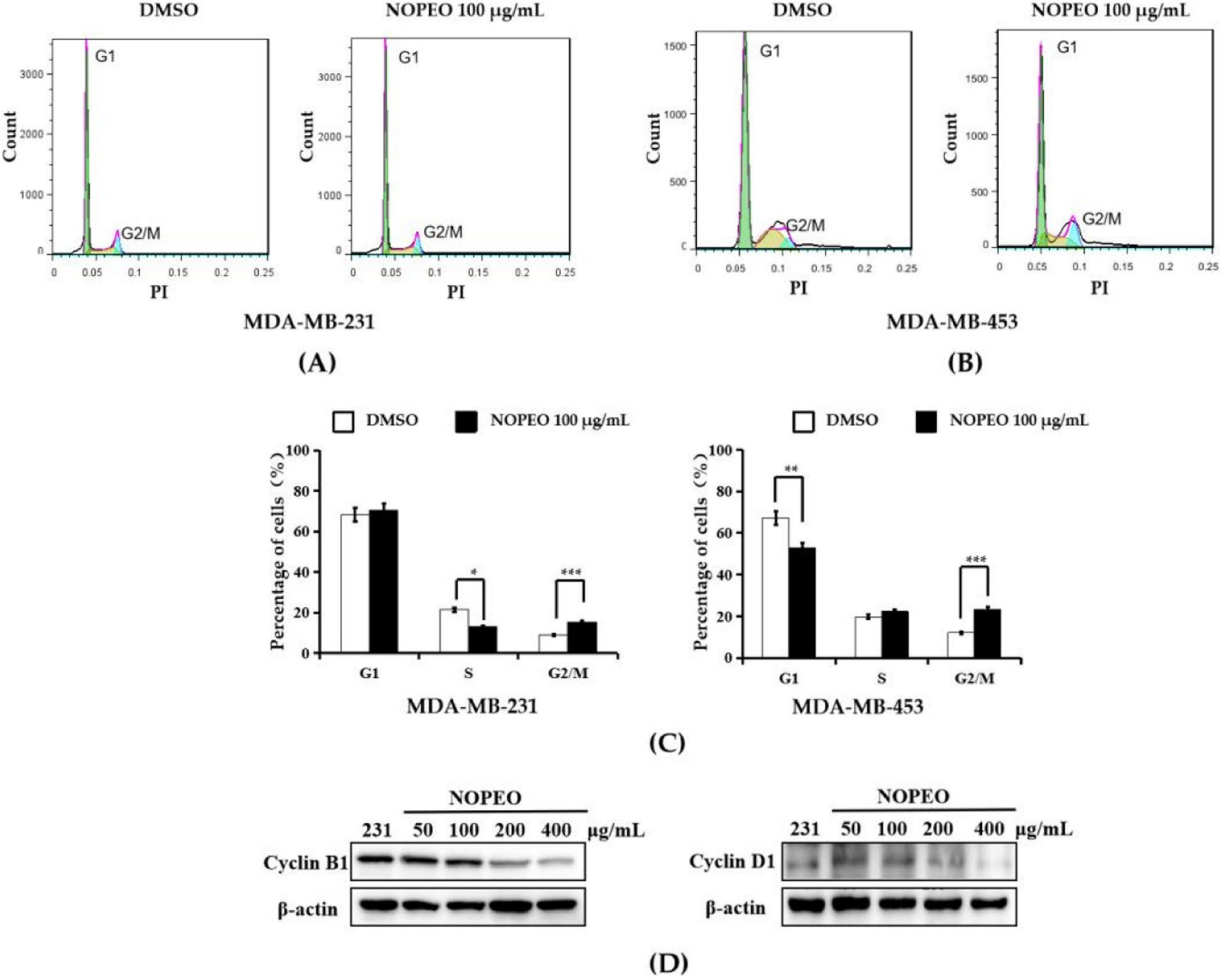
NOPEO blocked the cell cycle of TNBC cells. **A**-**B** TNBC cell cycle distribution. **C** Analysis of PI-stained DNA content by flow cytometry. ** *P* less than 0.01 and *** *P* less than 0.001 compared to the DMSO group. **D** The MDA-MB-231 cells were treated with 50–400 μg/mL NOPEO. The levels of Cyclin B1 and Cyclin D1 proteins were analyzed by western blotting

Treatment with NOPEO suppressed the cell growth of TNBC cells, but the molecular mechanism of this was not known. To determine whether NOPEO affected the cell cycle, the distribution of cell cycle was measured by PI staining. A NOPEO induced cell cycle inhibition was found after the treatment of TNBC cells for 24 h with 100 µg/mL NOPEO (Fig. 2A-B). TNBC cells showed an increased percentage of G2/M-phase cells (Fig. 2C). Uncontrolled cell growth is one of the characteristics of cancer and proper functioning of the cell cycle ensures that cells continue to proliferate. In this process, the proper function of cell cycle related proteins, including cyclinB1 and cyclinD1, is to ensure cell cycle proceeding correctly [25, 26]. In the NOPEO treated MDA-MB-231 cells, the protein levels of CyclinB1 and CyclinD1 were decreased (Fig. 2D).

### NOPEO induced the apoptosis of TNBC cells

**Fig. 3:**
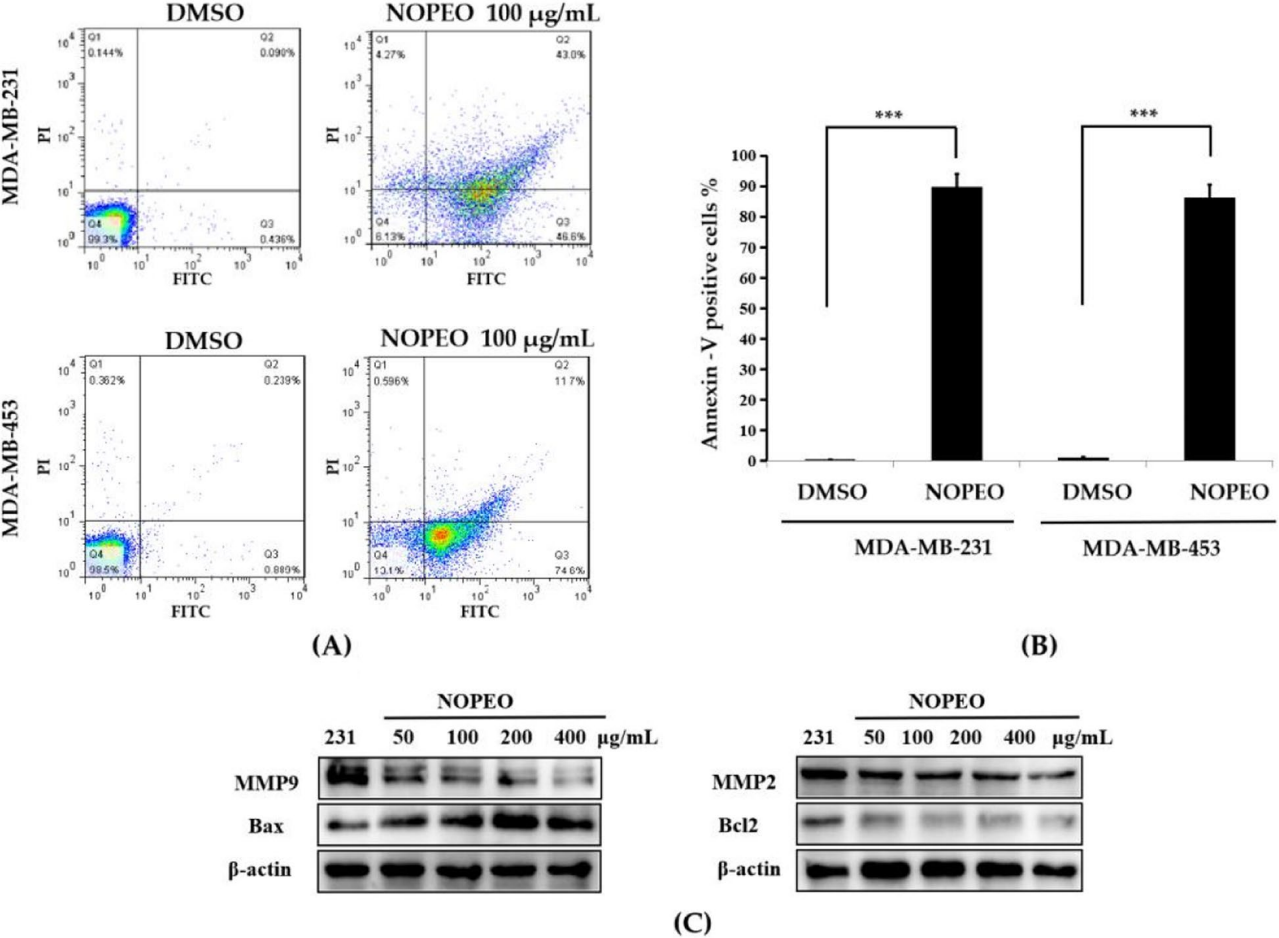
NOPEO induced cell apoptosis. **A** 100 μg/mL NOPEO were added toTNBC cells for 24 h and the rate of cell apoptosis were measured by flow cytometry. DMSO group as a negative control. Q1: death cells, Q2: late apoptosis, Q3: early apoptosis, Q4: living cells. **B** Columns represent the statistical result of the annexin V FITC/PI assay. *** *P* less than 0.001 compared with the DMSO group. **C** The MDA-MB-231 cells were treated with 50–400 μg/mL NOPEO. The levels of MMP9, MMP2, Bcl-2 and Bax protein were examined by western blotting

To further investigate the inhibiting effect of NOPEO on TNBC cell proliferation, we have examined the influence of NOPEO on cell apoptosis. TNBC cells were exposed to 100 μg/mL of NOPEO and used the Annexin-V FITC/PI assay to observe the rate of cell death. In Fig. 3, 46.6% of NOPEO treated MDA-MB-231 and only 0.436% of DMSO treated cells were in the early apoptotic phase. 74.6% of MDA-MB-453 cells in the NOPEO treatment group and only 0.889% of DMSO treated cells were in early apoptosis. The results indicated that NOPEO may be effective in inducing apoptosis and inhibiting proliferation in TNBC cells. Bcl-2 and Bax are the marker genes for the mitochondrial pathway of cell apoptosis, so when Bcl-2 expression is decreased and Bax is highly expressed, this means that apoptosis occurs through the mitochondrial pathway [27, 28]. The level of Bcl-2 was reduced after NOPEO treatment, and as the EO concentration increased, the expression of Bcl-2 showed a decreasing trend. On the contrary, there was an increase in Bax protein levels after handling with different densities of NOPEO, and the level of Bax expression tended to increase with increasing concentration of NOPEO (Fig. 3C). Matrix metalloproteinases (MMPs) are a class of zinc-dependent endopeptidases that degrade various protein components of the extracellular matrix (ECM). Members of this family have been implicated in cellular proliferation, migration and differentiation, angiogenesis, apoptosis and tissue repair. The expression of MMP2 and MMP9 are important indicators of the migration ability of tumour cells [29, 30]. In Fig. 3C, the levels of MMP2 and MMP9 protein decreased with increasing concentrations of NOPEO in MDA-MB-231 cells.

### NOPEO inhibited migration of the TNBC cells

**Fig. 4:**
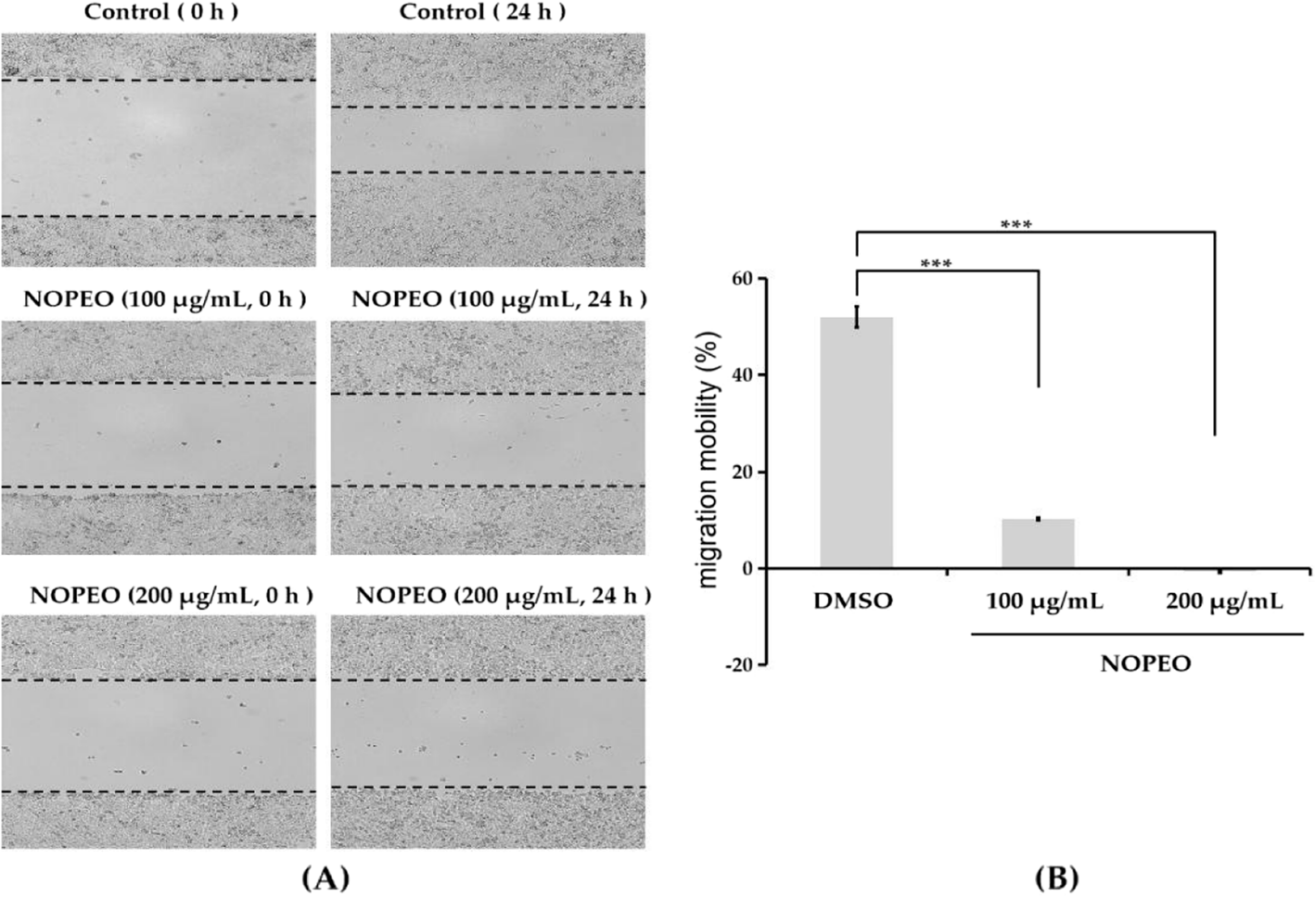
NOPEO regulated the migration of TNBC cells. **A** The migrate pictures of cells at 0 and 24 h. Serum-free medium was used as the control. Cell migration mobility was analyzed using Image J and Excel software. **B** ***P less than 0.001 indicates a significant difference

To examine the influence of NOPEO on the metastasis of TNBC, scratch wound trial was used to test the migration of TNBC cells. When the MDA-MB-231 cells were exposed to 100 μg/mL of NOPEO for 24 h, the cell migration was inhibited (Fig. 4A-B). The mobility rate of MDA-MB-231 cells in the presence of 200 µg/mL NOPEO was 0.61% ± 0.04%, which was 50 times lower than that of the control group(52.03% ± 2.14%).

### RNA-seq analyzed the differentially expressed genes of TNBC Cells treated by NOPEO

**Fig. 5:**
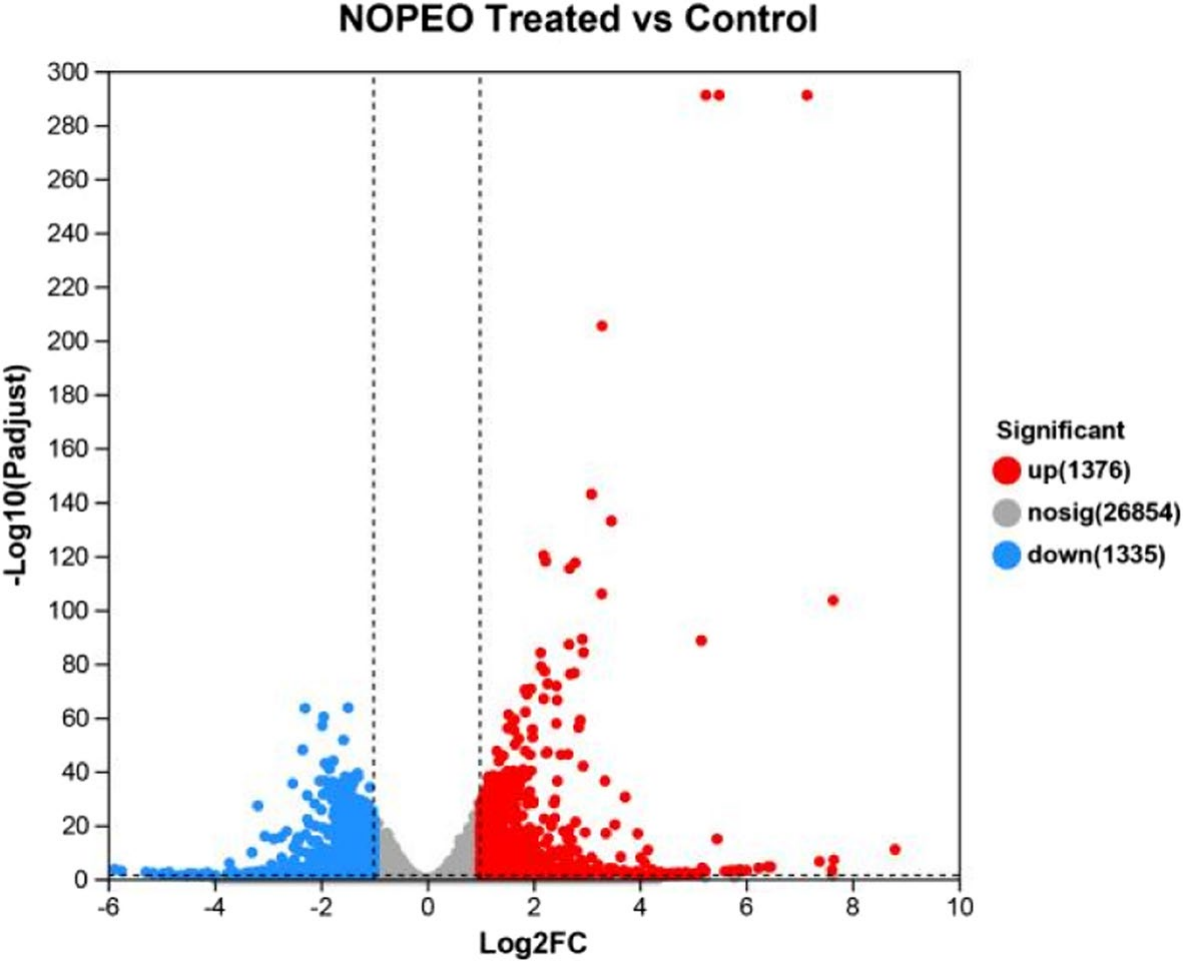
The Volcano plot of DEGs analyzed by RNA-seq. These genes are highlighted in colour and were found to be statistically significant (false discovery rate ≤ 0.05). Blue and red dots indicate significantly different down-regulated genes and up-regulated genes, respectively. Grey dots indicate genes that are not significantly different

**Fig. 6:**
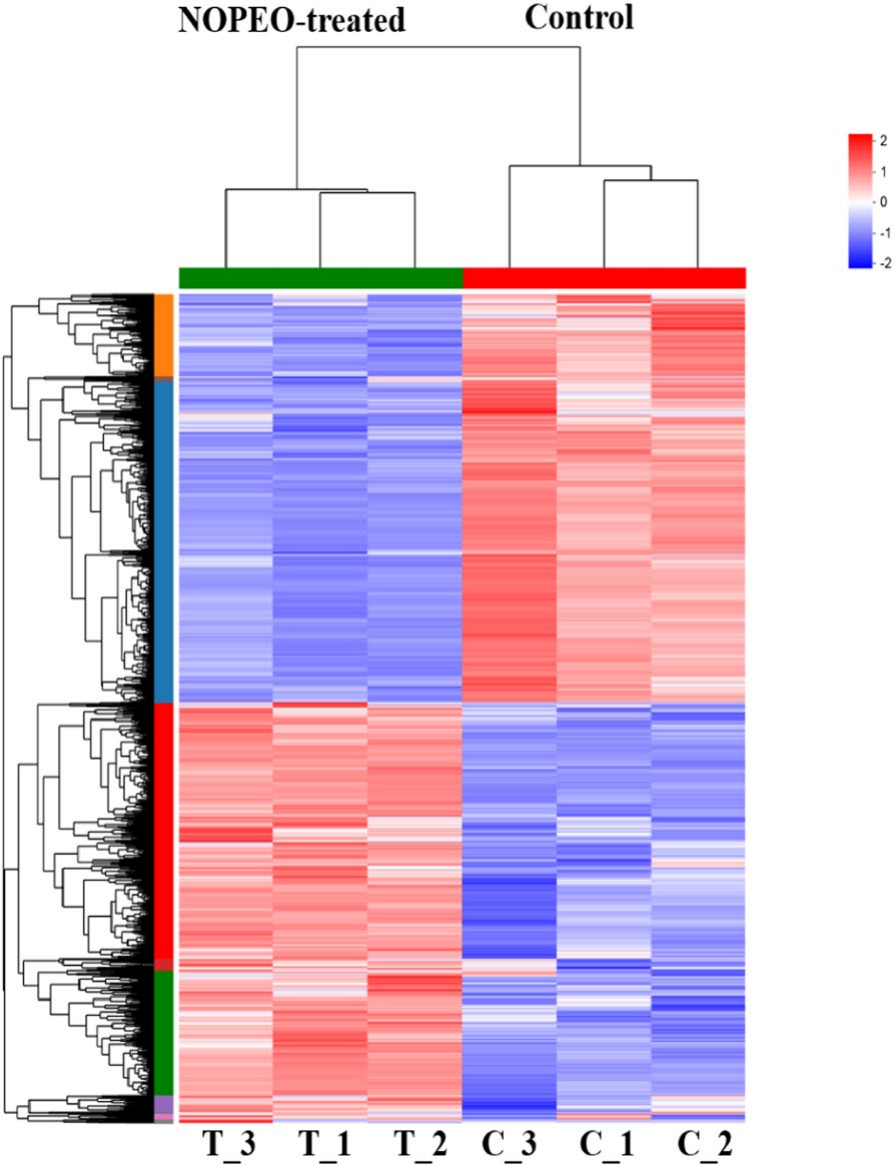
The Heat map of DEGs. The NOPEO treated group or control group were all replicated three times (*n* = 3). Genes were represented by rows, while samples were represented by columns. High expression relative to control cells was depicted in red blocks, while low expression was depicted in blue blocks

NOPEO could suppress the growth of TNBC cells by blocking cell cycle and promoting apoptosis, but the mechanism was unclear. MDA-MB-231 cells supplemented with 50 μg/mL of NOPEO were detected by RNA-seq array to investigate the mechanism of NOPEO in TNBC cells. RNA sequencing analysis revealed 2711 differentially expressed genes (DEGs) (1376 upregulated and 1335 downregulated) in experimental groups compared to control (Figs. 5 and 6). The top 10 DEGs are displayed in Table 1.

**Table 1:** Top 10 DEGs in MDA-MB-231 cells

**Rank**	**Gene Name**	**Gene Feature**	**log2C**	**Fold Change**	***p*-Value**
1	HSPA1B	Up-regulated	5.249906697	38.052	1.33E-295
2	SESN2	Up-regulated	3.292975006	9.801	1.36E-209
3	EGR1	Up-regulated	3.095641886	8.548	4.01E-147
4	DNAJB1	Up-regulated	3.471010767	11.089	3.97E-137
5	CDKN1A	Up-regulated	2.196698447	4.584	2.31E-124
6	PMEPA1	Up-regulated	2.231950652	4.698	4.12E-122
7	DUSP1	Up-regulated	2.789681817	6.915	1.83E-121
8	DDIT4	Up-regulated	2.684271355	6.428	2.57E-119
9	TOP2A	Down-regulated	-1.451776078	0.366	2.44E-29
10	ASPM	Down-regulated	-1.916514447	0.265	1.72E-39

### Analysis of function and pathway enrichment

**Fig. 7:**
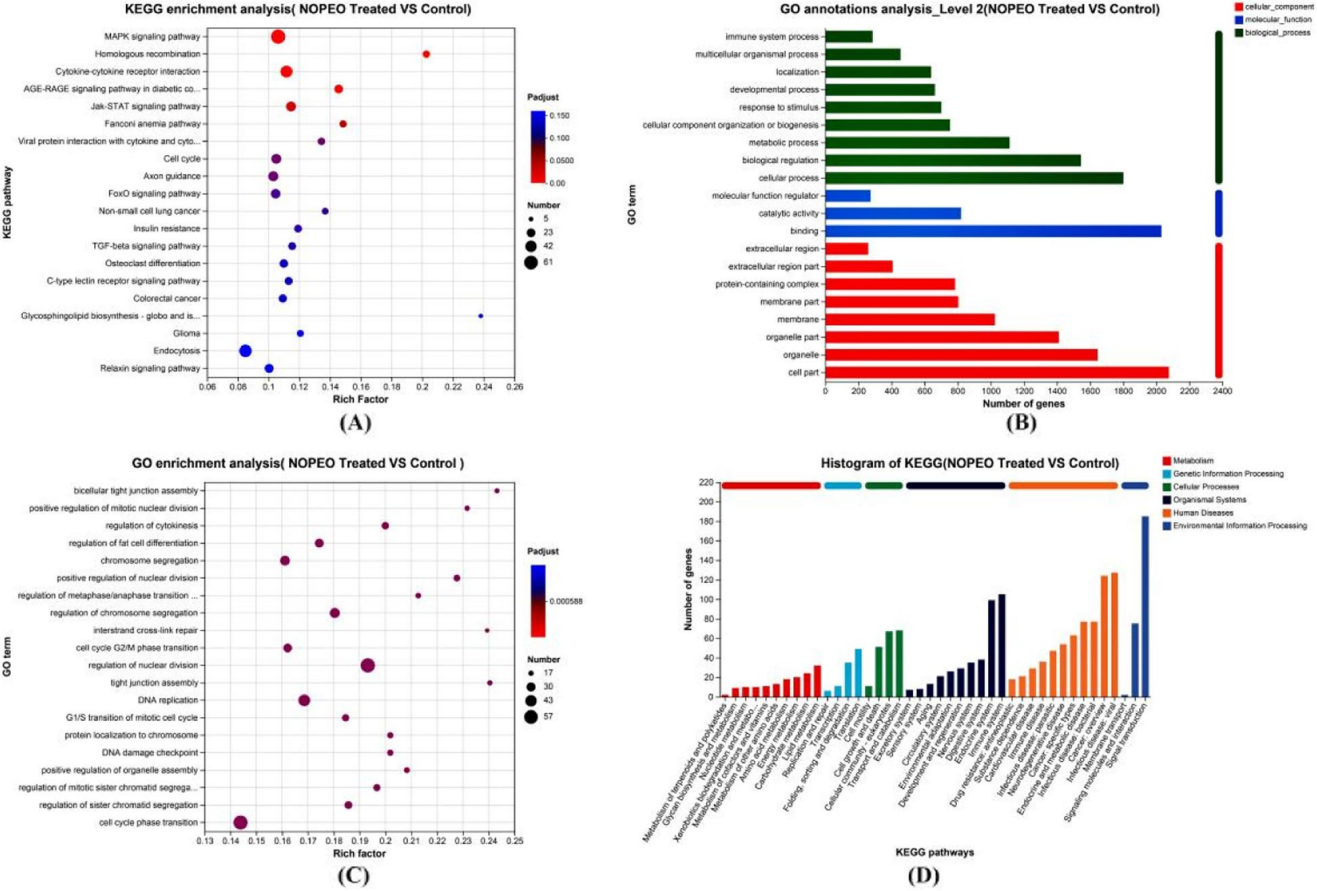
The function and signaling pathway which DEGs involved in analyzed by GO and KEGG. **A**-**B** The GO functional analysis of DEGs. **C**-**D** The KEGG analysis of DEGs

**Fig. 8:**
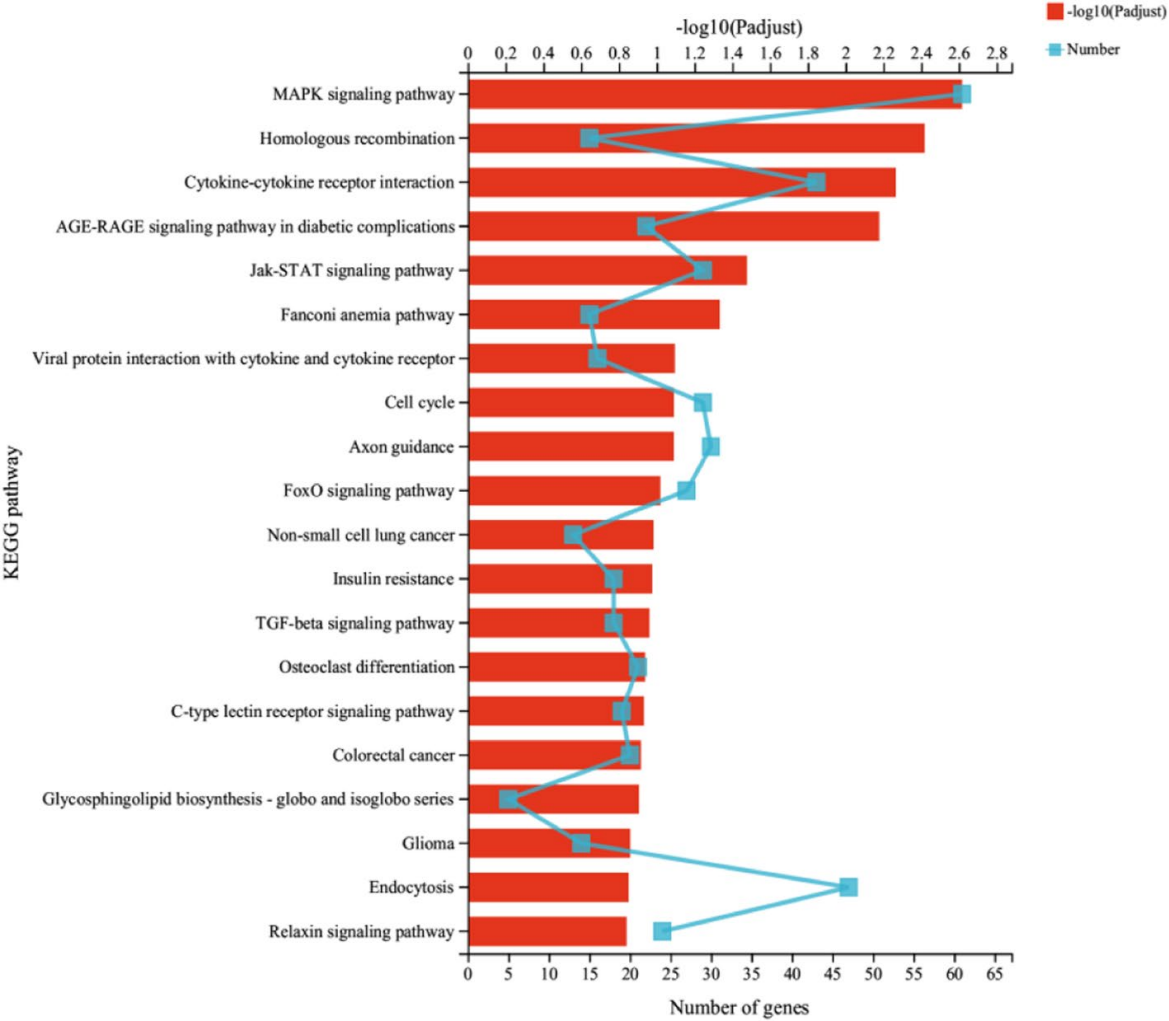
DEGs enrichment analysis for cell signalling pathways

**Fig. 9:**
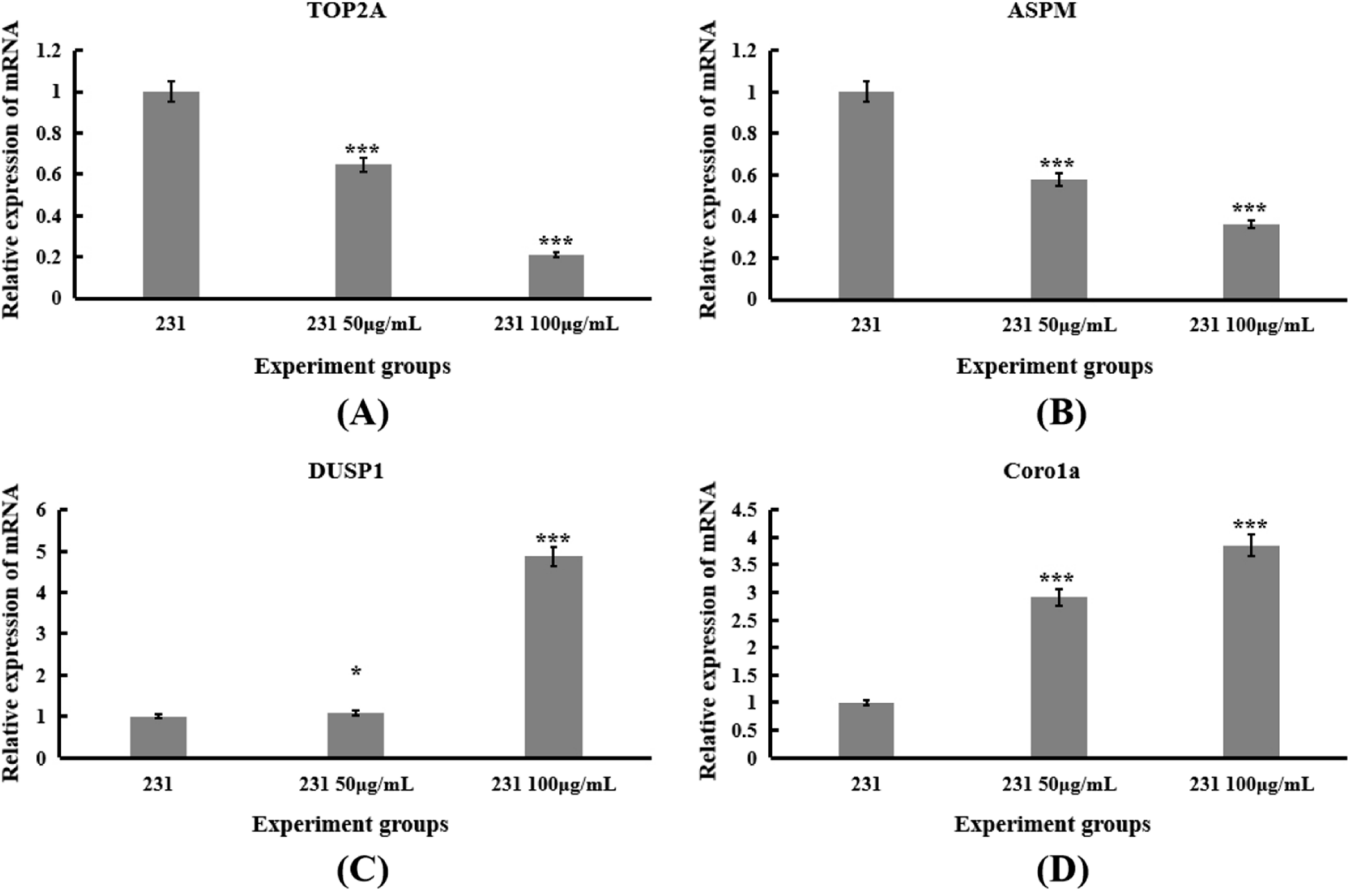
The changes of mRNA levels of DEGs were consistent with RNA-seq analysis. **A**-**D** The expression of ASPM, TOP2A, DUSP1 and Coro1a in NOPEO treated MDA-MB-231 cells and control cells. *** *P* less than 0.001 compared with the control group

The GO and KEGG analysis were used to confirm the primary functions of NOPEO on MDA-MB-231 cells, the significant difference in the results were displayed in Fig. 7. On the basis of the KEGG pathway results, we found that the changes in the MAPK and Jak/stat pathways were significant. HSPA1B, SESN2, EGR1, DNAJB1, CDKN1A, PMEPA1, DUSP1 and Coroa1 were up-regulated; TOP2A and ASPM were down-regulated (*p* < 0.05). KEGG analysis of the DEG genes revealed that 26 genes were associated with the MAPK pathway and 15 genes were involved in the transduction process of the Jak/stat pathway (Fig. 8). We speculated that NOPEO suppressed the growth of MDA-MB-231 cells by regulating the MAPK and Jak/stat signalling pathways [31]. Next, we verified the mRNA levels of TOP2A, ASPM, DUSP1 and Coroa1 genes expression on NOPEO treated cells or control by qPCR (Fig. 9). The qPCR results were consistent with the experimental data from RNA-seq.

## Discussion

In this research, we prepared NOPEO from the peel of navel oranges by cold pressing and molecular distillation. NOPEO exhibited significant inhibitory effects on cell viability, cell cycle distribution, cell apoptosis and migration of TNBC cell line MDA-MB-231 and MDA-MB-453. Our results indicate that exposure to NOPEO at concentrations up to 200 µg/mL results in a marked reduction in cell proliferation and migration, with the viability and mobility rate being reduced compared to the control group. This observation is consistent with the proposed role of NOPEO in suppressing TNBC cell growth.

To gain insights into the underlying mechanisms of NOPEO's action, we used RNA-seq analysis to profile the differentially expressed genes (DEGs) in MDA-MB-231 cells treated with NOPEO. A total of 2711 DEGs were identified, with approximately equal numbers being up-regulated and down-regulated. Functional and pathway enrichment analyses revealed that NOPEO primarily affected the MAPK and Jak/stat signalling pathways in MDA-MB-231 cells. Specific genes, such as HSPA1B, SESN2, EGR1, DNAJB1, CDKN1A, PMEPA1, DUSP1 and Coroa1 were up-regulated, while TOP2A and ASPM were down-regulated. The involvement of the MAPK and JAK/STAT pathways in cancer cell growth and proliferation is well-established, indicating that they will be prime targets for anticancer drug discovery [32, 33].

Of particular interest are the genes DUSP1 and Coroa1, which are known regulators of the MAPK pathway. DUSP1 encodes a dual-specificity phosphatase that dephosphorylates and thereby inactivates MAP kinases, thus suppressing cell proliferation and migration [34]. Coro1A, on the other hand, has been shown to promote cell death in certain cancer cells by activating apoptotic signalling [35]. The up-regulation of these genes in response to NOPEO treatment suggested that they may play a role in mediating the anti-proliferative effects of NOPEO in TNBC cells.

The down-regulation of TOP2A and ASPM genes is also noteworthy. TOP2A encodes topoisomerase II alpha, a key enzyme involved in DNA replication and mitosis. Its down-regulation can lead to impaired DNA replication and mitosis, ultimately resulting in cell cycle arrest and apoptosis [36]. ASPM, on the other hand, is a microtubule-associated protein that plays a role in mitotic spindle formation. Its downregulation is associated with mitotic defects and cell cycle arrest [37].

To validate the RNA-seq data, we performed qPCR to assess the mRNA levels of selected genes (TOP2A, ASPM, DUSP1, and Coroa1) in both NOPEO-treated and control cells. The qPCR results were consistent with the RNA-seq data, confirming the reliability of our findings.

Our study demonstrated that NOPEO effectively inhibited the migration of TNBC cells, possibly through the regulation of key genes and signalling pathways involved in cell cycle control and apoptosis. The identification of specific DEGs and their associated signalling pathways provided valuable insights into the molecular mechanisms underlying NOPEO's antiproliferative effects in TNBC cells. Future studies are warranted to further elucidate the therapeutic potential of NOPEO in TNBC treatment.

## Conclusions

Our research showed that NOPEO could induce cell cycle block and apoptosis by regulating the cell cycle-related genes Cyclin D1, CyclinB1, and apoprosis-related genes Bcl-2, Bax in the TNBC cells. NOPEO treatment significantly reduced MDA-MB-231 cell motility and downregulated metastasis associated proteins such as MMP-2 and MMP-9. The results of RNA-seq assay implied one possibility that NOPEO may inhibit the progression of TNBC cells via the MAPK and Jak/stat signalling pathways. On the basis of these results, NOPEO may be a potential natural product for the chemotherapeutics of TNBC.

### Supplementary Information

Supplementary Material 1.

Supplementary Material 2.

## Data Availability

The datasets used and/or analyzed during the current study are available from the corresponding author upon reasonable request. The results of RNA-seq were submitted to GEO, the accession number is GSE253079(https://www.ncbi.nlm.nih.gov/geo/query/acc.cgi?acc=GSE253079).

## Abbreviations

NOPEO: Navel Orange Peel Essential Oil
TNBC: Triple Negative Breast Cancer
PBS: Phosphate buffered saline
L-15: Leibovitz’s L-15
FBS: Fetal bovine serum
DMSO: Dimethyl sulfoxide
MMP: Matrix metalloproteinase
PI: Propidium Iodide
ECL: Enhanced chemiluminescence
qPCR: Realtime fluorescence quantitative PCR
DEGs: Differentially expressed genes
GO: Gene Ontology
KEGG: Kyoto Encyclopedia of Genes and Genomes
SD: Standard deviation
