# Supplementary material for: Navel orange peel essential oil inhibits the growth and progression of triple negative breast cancer
Source: BMC Complement Med Ther. 2024 Jun 14;24:233. doi: 10.1186/s12906-024-04525-y (PMC11177363; doi:10.1186/s12906-024-04525-y)

**Table S1 The sequence of primer were used in qPCR.** The TOP2A, ASPM, DUSP1 and Coroa1 genes were detected by qPCR with the following primer sequences.

| Primer | sequence（5' to 3'） |
| --- | --- |
| TOP2A AS | GAAGTGTCACCATTGCAGCC |
| TOP2A FS | AGCCATTTCGACCACCTGTC |
| ASPM AS | AACGCCATCAGGAGAGAGAG |
| ASPM FS | CCTCCACATAGCCTGAATAAGTGA |
| DUSP1 AS | GGATACGAAGCGTTTTCGGC |
| DUSP1 FS | GGTTGTCCTCCACAGGGATG |
| Coroa1 AS | ACCCTGACACCAACATCGTC |
| Coroa1 FS | TGGAACAGGTCCGACTTTCG |

# Original image of protein immunoblotting experiment

## Figure 2 D

**The expression of Cyclin B1 and β-actin was analyzed by western blotting.**

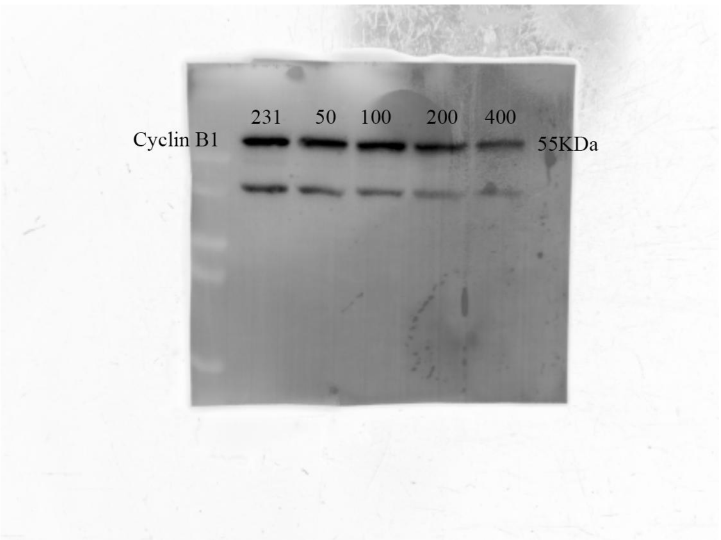

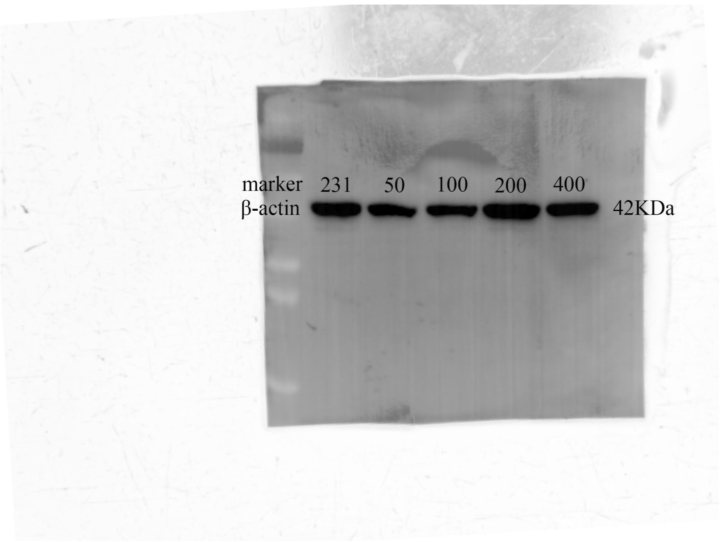
marker
231
50
100
200
400
β-actin
42KDa

**The expression of Cyclin D1 and β-actin was analyzed by western blotting.**

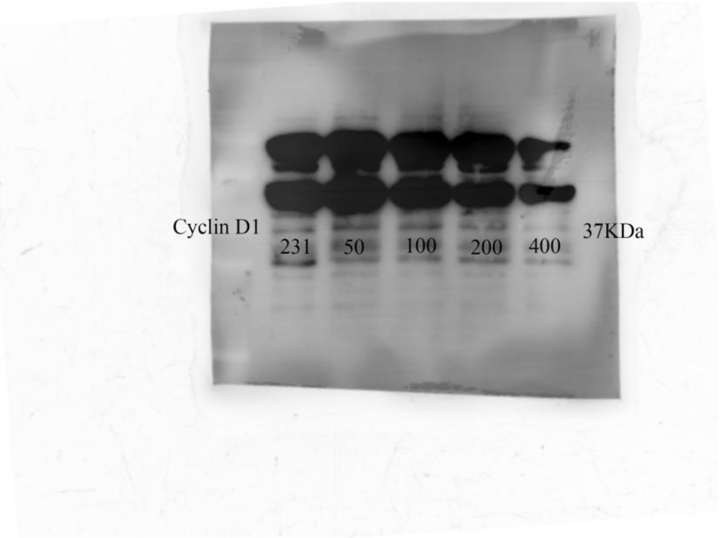


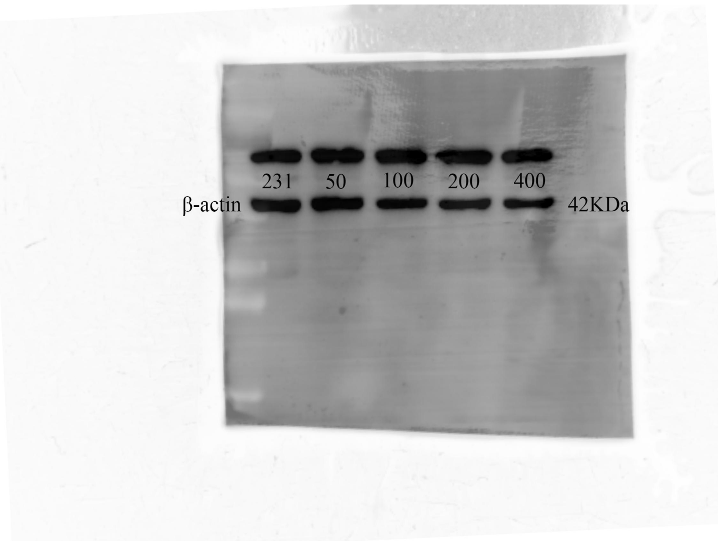

**Figure 3C**

**The expression of MMP9, BAX and β-actin was analyzed by western blotting.**

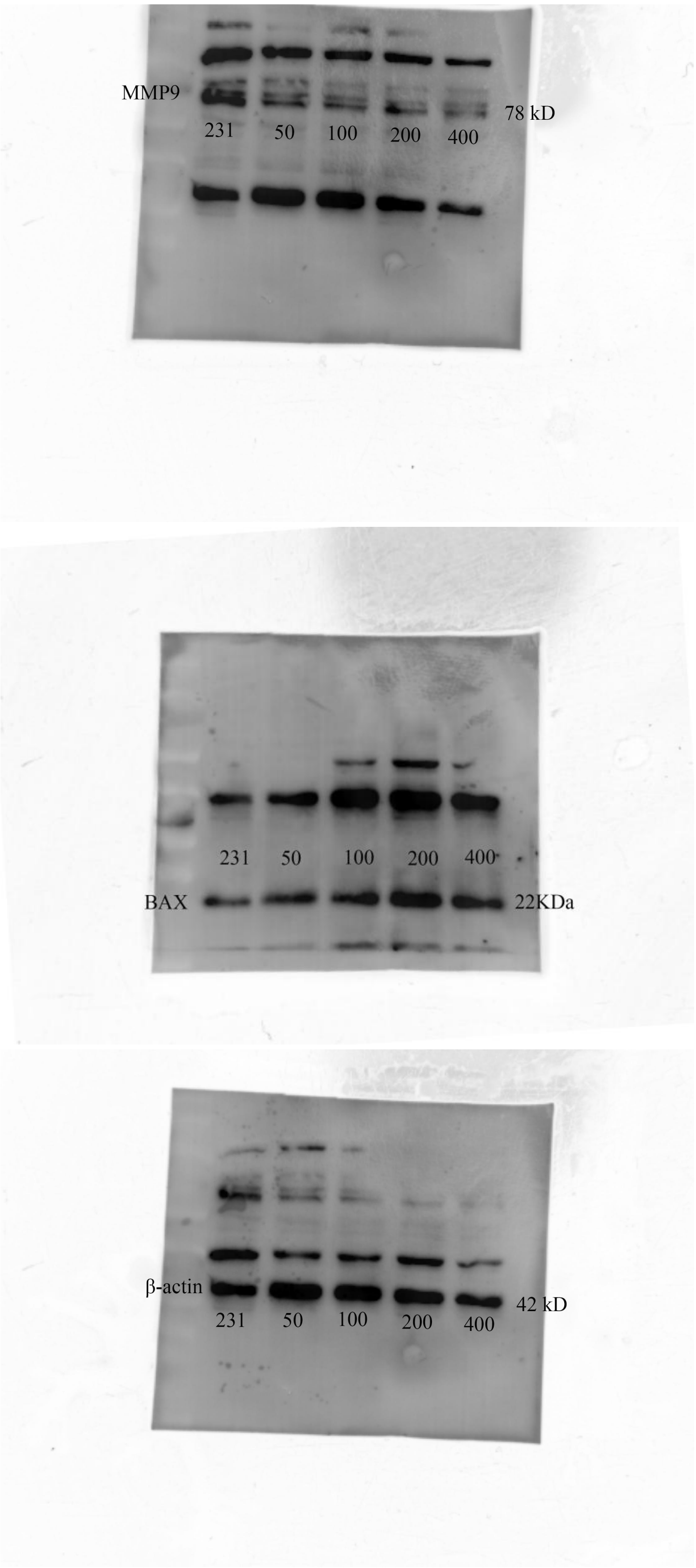

**The expression of MMP2, Bcl-2 and β-actin was analyzed by western blotting.**

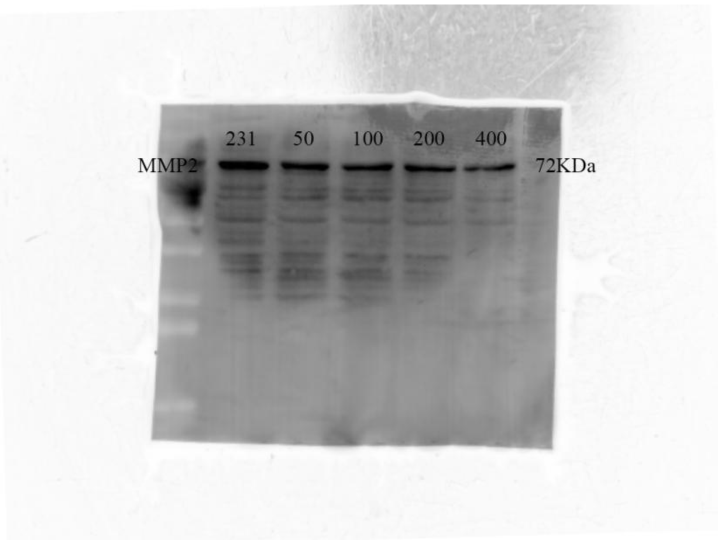


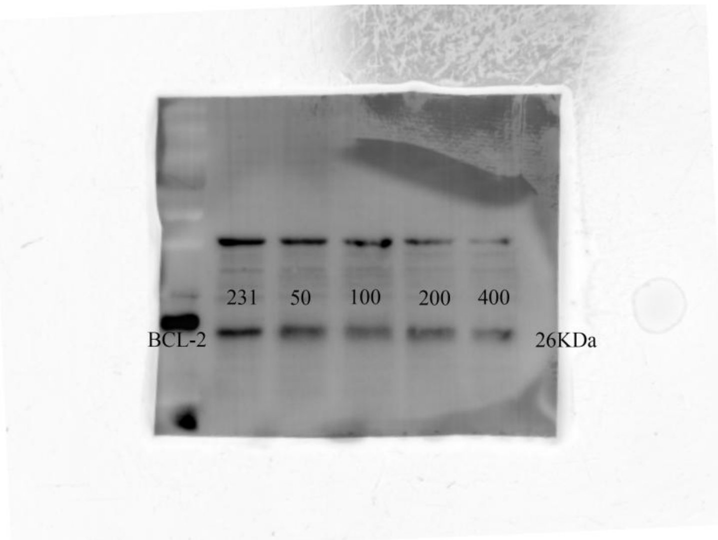


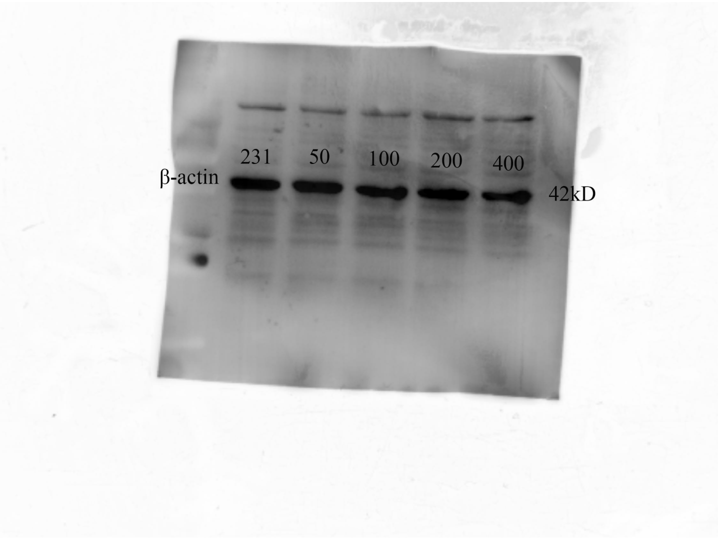

Supplement: Supplementary file 2 — Supplementary Material 2. [file 12906_2024_4525_MOESM2_ESM.pdf]
